# The *ezrin* Gene Regulates Early Cardiac Morphogenesis and Contractile Function in Zebrafish Through the Coordinated Regulation of Apoptosis, Calcium Homeostasis, and the MAPK Signaling Pathway

**DOI:** 10.3390/cells15121046

**Published:** 2026-06-07

**Authors:** Jinrui Lv, Ting Zeng, Beiya Liao, Ling Liu, Lei Xiong, Hao Xie, Lin Zhu, Xingzi Jiang, Zhuchuyu Zhong, Huaping Xie

**Affiliations:** 1 Hunan International Joint Laboratory of Animal Intestinal Ecology and Health, Laboratory of Animal Nutrition and Human Health, College of Life Sciences, Hunan Normal University, Changsha 410081, China; ljr0122@hunnu.edu.cn (J.L.); liaobeiya0120@hunnu.edu.cn (B.L.); 202120142637@hunnu.edu.cn (L.L.); xl@hunnu.edu.cn (L.X.); haox@hunnu.edu.cn (H.X.); 202420142862@hunnu.edu.cn (L.Z.); 202330194076@hunnu.edu.cn (X.J.); 202530233018@hunnu.edu.cn (Z.Z.); 2 Hunan Provincial Key Laboratory of Animal Intestinal Function and Regulation, Hunan Normal University, Changsha 410081, China; 3 Key Laboratory of Brain and Neuroendocrine Diseases, Hunan University of Medicine, Huaihua 418000, China; tingz@hunnu.edu.cn

**Keywords:** zebrafish, *ezrin*, heart development, myocardial contraction, morpholino

## Abstract

**Highlights:**

**What is the main finding?**

- No overt developmental abnormalities were observed in *ezra*^−/−^, where only *ezra* was knocked out. The simultaneous deficiency of the *ezra* and *ezrb* genes resulted in pericardial edema, reduced cardiac chamber size, and atrioventricular valve developmental defects in embryos, accompanied by a decreased heart rate.

**What is the implication of the main finding?**

- Functional redundancy was demonstrated between *ezra* and *ezrb*, and *ezrin* plays a critical role in cardiac morphogenesis and functional maintenance.

**Abstract:**

Ezrin, expressed by the *EZR* gene, is a member of the ERM protein family that connects the plasma membrane to the actin cytoskeleton, participating in processes such as cell adhesion, migration, and signaling. However, its role in cardiac morphogenesis remains incompletely understood. In zebrafish (*Danio rerio*), two *ezrin* homologs, *ezra* and *ezrb*, are present. CRISPR/Cas9 gene editing technology was used to generate *ezra* knockout lines, and the simultaneous knockdown of *ezra* and *ezrb* was induced via morpholino oligonucleotides (MOs). To investigate the molecular mechanisms, transcriptome sequencing and bioinformatic analysis were conducted on 48 h post-fertilization (hpf) *ezrin*–MO embryos, with subsequent validation using a real-time quantitative polymerase chain reaction (RT-qPCR) and whole-mount in situ hybridization (WISH) experiment. The results showed that *ezra*^−/−^ exhibited a compensatory upregulation of *ezrb* without overt developmental defects, whereas *ezrin*–MO embryos presented with pericardial edema, reduced cardiac chamber size, and atrioventricular valve malformations at 48 hpf. RNA-seq revealed that myocardial contraction-related genes were significantly dysregulated and apoptotic signaling pathways were activated in *ezrin*–MO embryos. These findings demonstrate that *ezra* and *ezrb* are functionally redundant in cardiac development and that the loss of *ezrin* function may lead to cardiac developmental defects and impaired myocardial contractility via the activation of apoptotic signaling pathways.

## 1. Introduction

During vertebrate organogenesis, the heart is the first organ to form and function [1]. Zebrafish (*Danio rerio*) heart development is initiated by the directed migration and aggregation of mesodermal cells from the anterior lateral plate, resulting in the formation of the primitive heart tube [2,3]. Subsequently, the heart tube is driven to form a functional organ by the coordinated action of cells from the first heart field (FHF) and the second heart field (SHF) [4]. This process can be divided into several sequential stages, including precursor cell migration (5–12 h post-fertilization, hpf) [5], heart tube assembly (15 hpf) and fusion (approximately 18 hpf) [6], cardiac chamber formation (approximately 22 hpf), linear heart tube formation (24 hpf) [7], and heart tube looping (post-24 hpf) [8], with looping largely complete by 48 hpf.

At approximately 24–30 hpf, the zebrafish heart presents as a double-layered, linear tubular structure [9], characterized by the separation of the endocardium and myocardium via the cardiac extracellular matrix (ECM) [10], which plays a critical role in extracellular signaling and cardiomyocyte migration [11]. At 37 hpf, atrioventricular valve formation initiates in zebrafish embryos; concurrently, the expression of *bmp4* and *versican* is restricted to the myocardium of the atrioventricular canal (AVC) [12]. Following the dextral looping of the heart tube, the ECM within the AVC and outflow tract (OFT) regions undergoes localized thickening, and the junctional state between activated endocardial cells (EdCs) is altered, resulting in their separation [13]. Regulated by signaling molecules including *TGFβ*, *bmp2/4/5* and *notch1-4*, endocardial cells undergo an epithelial–mesenchymal transition (EMT) [14]. These cells then migrate into the cardiac stroma, where the proliferation and secretion of extracellular matrix components occur to form the endocardial pad, which establishes the foundation for the subsequent morphogenesis of the atrioventricular valves [15,16]. Owing to its embryonic transparency [17,18], conserved cardiac development [19], facile genetic manipulation [20], and viability during early developmental stages even with severe cardiac defects [21], the zebrafish serves as an ideal model organism for investigating the mechanisms of early heart development.

Ezrin, encoded by the *EZR* gene, belongs to the highly conserved Ezrin/Radixin/Moesin (ERM) protein family [22]. Ezrin is composed of an N-terminal Four-point-one, Ezrin, Radixin, Moesin (FERM) domain; an intermediate α-helical domain; and a C-terminal ERM-associated domain (C-ERMAD) [23,24]. In its resting state, Ezrin maintains a closed conformation. Activation occurs via phosphorylation (e.g., by signaling molecules including PKC and Rho kinases at the Thr567 residue) [25,26], mediating the linkage between the plasma membrane and the cytoskeleton. Subsequent dephosphorylation results in reversion to the closed state. Furthermore, its activity is modulated by lipid molecules such as phosphatidylinositol 4,5-bisphosphate (PI(4,5)P_2_) and post-translational modifications, specifically acetylation and ubiquitination [27,28,29].

The Ezrin protein is primarily localized to submembrane regions, including microvilli, membrane folds, and pseudopods [30], where it functions as a “scaffold” by creating complexes of membrane proteins, Ezrin, and the cytoskeleton, and is involved in processes such as cell signaling, morphogenesis, and migration. Interactions between Ezrin and cell adhesion molecules, including CD44 and L-selectin, mediate their cross-linking with actin filaments and facilitate signal transduction between the cytoskeleton and adhesion proteins [31,32]. Ezrin promotes the formation of pseudopods during cell migration and invasion by regulating cytoskeletal reorganization, and it activates the RhoA/ROCK signaling pathway to enhance cell migration capacity [33,34,35]. During the epithelial–mesenchymal transition (EMT), Ezrin facilitates the depolarization of polarized epithelial cells and their conversion into mesenchymal cells [36]. Extensive tumor biology research has demonstrated that the *EZR* gene is aberrantly overexpressed in various cancer cells [37] and is intimately associated with tumor cell invasion and metastasis [38]. Furthermore, its function in immune regulation [39,40,41,42], modulation of B-cell immune responses [43], and initiation of B-cell receptor (BCR) activation [44,45,46,47]—whereby Ezrin deficiency ameliorates lupus phenotype via reduced B-cell activation levels [48]—along with its contribution to the morphogenesis of specialized structures including microvilli, inner ear cilia and primary cilia [49,50,51,52,53,54], has been extensively validated. Nevertheless, the specific function of Ezrin in cardiac development remains to be elucidated.

Ezrin is linked to the establishment of planar cell polarity (PCP) in previous studies. During zebrafish gastrulation, a marked reduction in total EZRB protein abundance and Thr567 phosphorylation is observed following the loss of *vangl2* function. Consequently, the connection between the plasma membrane and cortical actin is attenuated, leading to the induction of vesicular protrusions in mesodermal and endodermal cells during late gastrulation and the inhibition of PCP establishment and directed migration [55]. Furthermore, zebrafish *vangl2* homozygous mutants are characterized by the abnormal migration of cardiac precursor cells in the FHF, resulting in cardiac cleavage (the appearance of two short cardiac tubes at the midline), abnormal cardiac tube positioning [56], and looping defects [57]. In addition, as an effector of PCP signaling, *vangl2* regulates outflow tract elongation through maintaining the polarity and epithelial characteristics of SHF cells [58]. Collectively, these findings suggest the potential involvement of Ezrin in early cardiac morphogenesis. *EZR* has been identified as a candidate gene integral to cardiac development through prior screening by our laboratory [59]. The zebrafish genome presents two *ezrin* paralogs, *ezrin a* (*ezra*) and *ezrin b* (*ezrb*), which are hypothesized to have functional redundancy during development. However, the specific functions of *ezrin* in cardiac development and functional regulation warrant further investigation.

To investigate the function of *ezrin* in cardiac development, an *ezra* knockout line and *ezrin*–MO embryos were generated using CRISPR/Cas9 gene editing and morpholino oligonucleotide knockdown, respectively. The RT-qPCR experiment results indicated that *ezra* knockout resulted in the upregulated expression of *ezrb* mRNA, and no obvious developmental defects were observed in embryogenesis, implying potential functional redundancy between these two genes. Next, *ezrin*–MO embryos displayed pericardial edema and diminished cardiac chamber size at 48 hpf, concomitant with valvular developmental defects. Further transcriptomic profiling and bioinformatic analysis were conducted on 48 hpf double-knockdown embryos. The results indicated that *ezrin* deficiency led to the aberrant activation of the apoptosis pathway and dysregulated expression of genes involved in myocardial contraction, implying that *ezrin* is implicated in early cardiac morphogenesis and the regulation of cardiac function in zebrafish embryos.

In summary, due to the high conservation of cardiac gene expression between zebrafish and humans (approximately 96% of cardiomyopathy-associated genes exhibit conserved expression [60]) and the notable regenerative capacity of zebrafish cardiac muscle [61,62], the role of *ezrin* in early cardiac morphogenesis and functional regulation was preliminarily elucidated. Furthermore, this paper offers a novel theoretical framework for the in-depth investigation of regulatory networks that affect cardiac development and for the modeling of human congenital heart abnormalities.

## 2. Materials and Methods

### 2.1. Zebrafish Breeding and Ethics

Tuebingen (TU), TL (*nkx2.5*:ZsYellow), Tg(*kdrl*:DsRed), and Tg(*fli-1a*:EGFP) zebrafish were provided by the Laboratory of Animal Nutrition and Human Health, College of Life Science, Hunan Normal University. Tg(*fli*-1a:EGFP) zebrafish were incrossed to generate embryos that express green fluorescence specifically in vascular endothelial cells. TL (*nkx2.5*:ZsYellow) were crossed with Tg(*kdrl*:DsRed) zebrafish to generate embryos that simultaneously expressed heart-specific green fluorescence and vascular-specific red fluorescence.

Embryos were cultured under the following conditions: the water temperature was 28.0 ± 0.5 °C, the pH was 6.5–7.5, salinity was 450–500 μS/cm, and the photoperiod was 14 h of light and 10 h of darkness. E3 solution was used to cultivate embryos at 28.5 °C. At the one-cell stage, micro-injection was carried out. All experiments were conducted in compliance with the guidelines authorized by the Hunan Normal University Animal Ethics Committee.

### 2.2. Generation of Zebrafish ezra Knockout Lines

The *ezra* knockout zebrafish lines were generated by CRISPR/Cas9 gene editing technology [63]. The mRNA and protein sequences of the *ezra* gene were retrieved from the NCBI database. The protein domain of EZRA was analyzed with the SMART online tool. The target sites for *ezra* were determined through the CRISPOR website and were situated inside exon 4. Two target sites were accessed: sequence 1 (*ezra*-sgRNA-F1) is 5′-GCGTAATACGACTCACTATAGGTGTCAGAGGAACTGATTCGTTTTAGAGCTAGAAATAG-3′, and sequence 2 (*ezra*-sgRNA-F2) is 5′-GCGTAATACGACTCACTATAGGACCTGAAACCCACAAAACGTTTTAGAGCTAGAAATAG-3′. The T7 promoter sequence was added to the 5′ terminus of each target sequence of the forward primers, and PCR was conducted utilizing the forward (*ezra*-sgRNA-F1/2) and reverse primer (sgRNA-R). The PCR products underwent DNA purification, transcription, and RNA purification to generate sgRNA. The purified sgRNAs were subsequently mixed with the Cas9 protein (ThermoFisher, Waltham, MA, USA) for micro-injection. Genomic DNA was retrieved from F0 embryos, and *ezra* mutations were identified using the genotyping primers *ezra*-GT-F (5′-AGTTTAAGTTTCGGGCCAAGC-3′) and *ezra*-GT-R (5′-TTATGATGAAGCGGGGTTGG-3′). To assess sgRNAs’ efficiency, injected and wild-type (WT) embryos were randomly chosen at 36 hpf. The WT amplicon measured 500 bp, and the two target sites were separated by 130 bp. If both sites of genomic DNA were edited effectively, a deletion of ~130 bp or a randomized insertion was observed, compared to the control. Subsequent to validating the sgRNAs’ efficiency, the remaining F0 embryos were raised to 45 dpf. Fish exhibiting DNA deletions or insertions were selected and crossed with the WT to generate F1. Genotyping was conducted on F1 individuals, and DNA fragments under 500 bp were extracted and submitted for Sanger sequencing. Homozygous *ezra* mutants (F2 generation, *ezra*^−/−^) were used for subsequent experiments.

### 2.3. Morpholino-Induced Targeted Knockdown of ezra and ezrb Genes

Morpholino oligonucleotides directed against *ezra* and *ezrb* were acquired from Gene Tools (Philomath, OR, USA) to block protein translation (*ezra*–MO: 5′-ACATTGATAGGCTTCGGCATTGTGA-3′; *ezrb*–MO: 5′-TTTTGATGTAGATGCCGATTCCTCT-3′). MOs were injected into WT embryos at the one-cell stage. Embryonic development was monitored regularly, and the incidence of malformed phenotypes was statistically analyzed.

### 2.4. Real-Time Quantitative Polymerase Chain Reaction (RT-qPCR)

Three independent biological replicates, each comprising 50 embryos at 48 hpf, were collected for each group (WT, *ezra*^−/−^, and *ezrin*–MO). All samples were frozen in liquid nitrogen and stored at −80 °C. Total RNA was obtained from embryos with TRIZOL (Takara, Kusatsu, Japan) according to the manufacturer’s guidelines. Subsequently, 1 μg of total RNA was reverse-transcribed into cDNA, applying reverse transcriptase (Takara) and oligonucleotide primers. RT-qPCR experiment was conducted utilizing 2× SYBR Green Master qPCR Mix (Vazyme, Nanjing, China) on a QuantStudio 3 real-time PCR system. The primers used for RT-qPCR experiment in this investigation are enumerated in Table S1. The RT-qPCR experiment results were evaluated by the ΔCt (2−^ΔΔCt^) method.

### 2.5. Whole-Mount in Situ Hybridization (WISH)

The mRNA sequences of the target genes were acquired from NCBI, and primer sequences for antisense RNA probes were generated. Antisense RNA probes targeting the *cmlc1*, *cmlc2*, *nppa*, *gata4*, *nkx2.5*, *hand2*, *bmp4*, *notch1b*, *tnnt2a* and *tnni2b.1* genes were generated by in vitro transcription. For these genes, Table S2 contains the primer sequences. The specified developmental stages of the embryos were fixed in 4% paraformaldehyde (PFA) at 4 °C overnight, followed by two PBST washes, proteinase K treatment (10 mg/mL), post-fixation in 4% PFA, and PBST rinsing. After pre-hybridization for 1–4 h, the RNA probes were added to the embryos overnight at 68 °C. The second day, the embryos were incubated with anti-DIG antibody overnight after being successively rinsed with 50% formamide/2× SSCT, 2× SSCT, 0.2× SSCT, and MABT. Following three MABT washes, color development was carried out in the darkness at ambient temperature using the BCIP-NBT mixture. Using a Leica stereo microscope, images were captured.

### 2.6. RNA Sequencing (RNA-Seq) and Differential Expression Gene Analysis

Three biological replicates, each including 50 embryos at 48 hpf, were obtained for each group (WT and *ezrin*–MO). RNA sequencing was conducted by Shanghai Ouyi Biomedical Technology Co., Ltd. (Shanghai, China). The Agilent 2100 Bioanalyzer (Agilent Technologies, Inc., Santa Clara, CA, USA) was used to measure RNA integrity after total RNA was collected by the mirVana miRNA Separation Kit (Ambion, Waltham, MA, USA). Six cDNA libraries were established according to manufacturer guidelines and sequenced using the Illumina platform. Raw reads were processed via Trimmomatic and aligned to GRCz11 utilizing Hisat2. FPKM was derived from featureCounts output files. Genes exhibiting FPKM > 1 were deemed expressed. Thresholds of *p* < 0.05 and |log_2_FoldChange| > 1 were used to identify differentially expressed genes (DEGs). The distribution of DEGs was illustrated using volcano plots and heatmaps. Gene Ontology (GO) and Kyoto Encyclopedia of Genes and Genomes (KEGG) enrichment analyses were conducted utilizing the R package clusterProfiler (v4.8.3). The results were shown with the R package ggplot2 (v3.4.3) and the web platform Sangerbox 3.0. Furthermore, Gene Set Enrichment Analysis (GSEA) was carried out on the gene list prioritized by fold change. The enrichment in upregulated and downregulated gene sets in the KEGG pathways was assessed. Pathways with a normalized enrichment score (NES) > 0 were classified as upregulated, whereas those with an NES < 0 were classified as downregulated. biomaRt software (v3.2.0) was utilized to annotate key genes in each pathway. The GSEA results were illustrated using “GseaVis”.

### 2.7. Cardiac Physiological Analysis

The ventricular activity of zebrafish embryos at 48 hpf was recorded for 10 s at 150 frames s^−1^ using a high-speed EM-CCD camera under a 10× microscope (HAMAMATSU, Shizuoka-ken, Japan). The collected heartbeating movies were then analyzed with semi-automatic heartbeat analysis software (v 3.4.0.0) to obtain cardiac contraction-related parameters: heart rate (HR), heart period (HP), diastolic interval (DI), and systolic interval (SI). In addition, cross-sectional images were obtained from the heartbeat videos to visually illustrate the changes in the aforementioned cardiac cycle parameters. All data were also reflected in the generated M-mode images [64].

### 2.8. Statistical Analysis

All trials were conducted at least three times. Statistical analyses were performed using GraphPad Prism (v8.0.1). Significant differences across various embryo groups were evaluated by Student’s *t*-test.

## 3. Results

### 3.1. Spatiotemporal Expression of Zebrafish ezrin Gene

**Figure 1:**
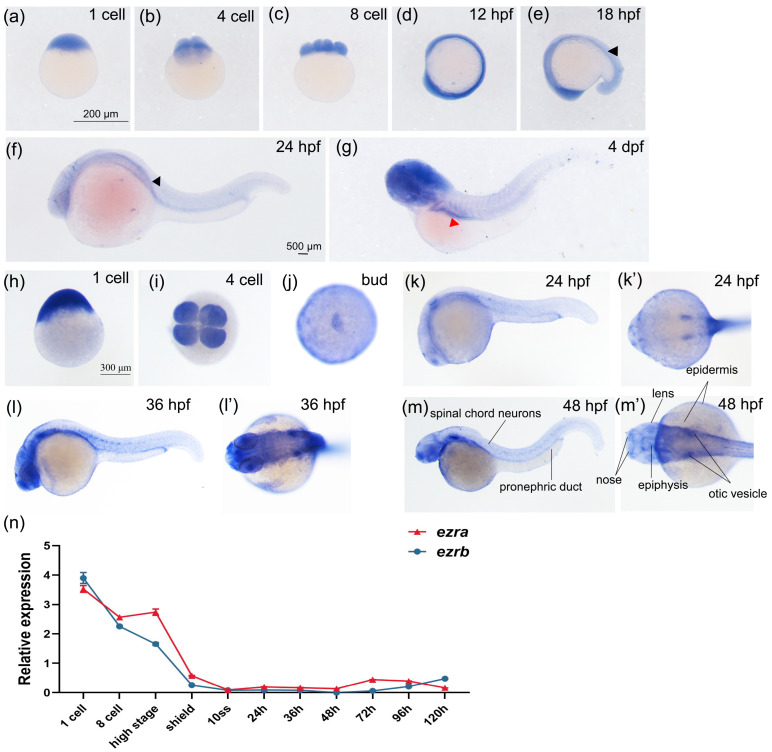
The spatiotemporal patterns of *ezra* and *ezrb* in zebrafish. The WISH experiment results of *ezra* (**a**–**g**) and *ezrb* (**h**–**m′**) expression. *ezra* was detected during the 1–8-cell stages (**a**–**c**), exhibited widespread expression at 12 hpf (**d**), and expressed in the notochord at 18 hpf (**e**) and 24 hpf (**f**) (black triangle), as well as the intestine at 4 dpf (red triangle); *ezrb* expression was detected during the 1–8-cell stages (**h**,**i**), ubiquitously expressed at the tail bud stage (**j**), and localized to the lens, auditory vesicle, nose, epidermis, epiphysis, renal ducts, and notochord neurons at 24 hpf (**k**,**k′**), 36 hpf (**l**,**l′**), and 48 hpf (**m**,**m′**). (**n**) The RT-qPCR experiment results of *ezra* and *ezrb* expression at different developmental stages, with ef1α used as the endogenous reference. In the RT-qPCR experiment, three independent biological replicates were established for the WT at each developmental time point, with each replicate containing 50 embryos at 48 hpf. Scale bar: 200 μm (**a**–**e**); scale bar: 500 μm (**f**,**g**); scale bar: 300 μm (**h**–**m′**).

To explore the potential functions of *ezra* and *ezrb* in zebrafish development, WISH and RT-qPCR experiments were employed to investigate the spatiotemporal expression patterns of these genes. The WISH experimental results showed that *ezra* expression was detected during the 1–8-cell stages, suggesting maternal expression (Figure 1a–c). At 12 hpf, *ezra* was ubiquitously expressed (Figure 1d). Subsequently, at 18 hpf and 24 hpf, *ezra* expression was restricted to the notochord (Figure 1e,f), and at 4 dpf (days post-fertilization), specific expression was observed in the intestine (Figure 1g). Similarly, *ezrb* exhibited maternal expression (Figure 1h–i). During the tail bud stage, *ezrb* displayed broad expression (Figure 1j). In embryos at 24 hpf, 36 hpf, and 48 hpf, *ezrb* expression was localized to the lens, auditory vesicle, nose, epidermis, epiphyses, renal ducts, and notochord neurons (Figure 1k–m′). The RT-qPCR experiment results were consistent with the WISH experimental results (Figure 1n). *ezra* and *ezrb* mRNA levels were abundant in one-cell embryos, followed by a gradual decline until the 10-somite (10 ss) stage, where expression plateaued at a low level. These findings suggest that *ezra* and *ezrb* may play critical roles in early embryogenesis.

### 3.2. Knockout of Zebrafish ezra Gene Does Not Affect Overall Embryonic Development

**Figure 2:**
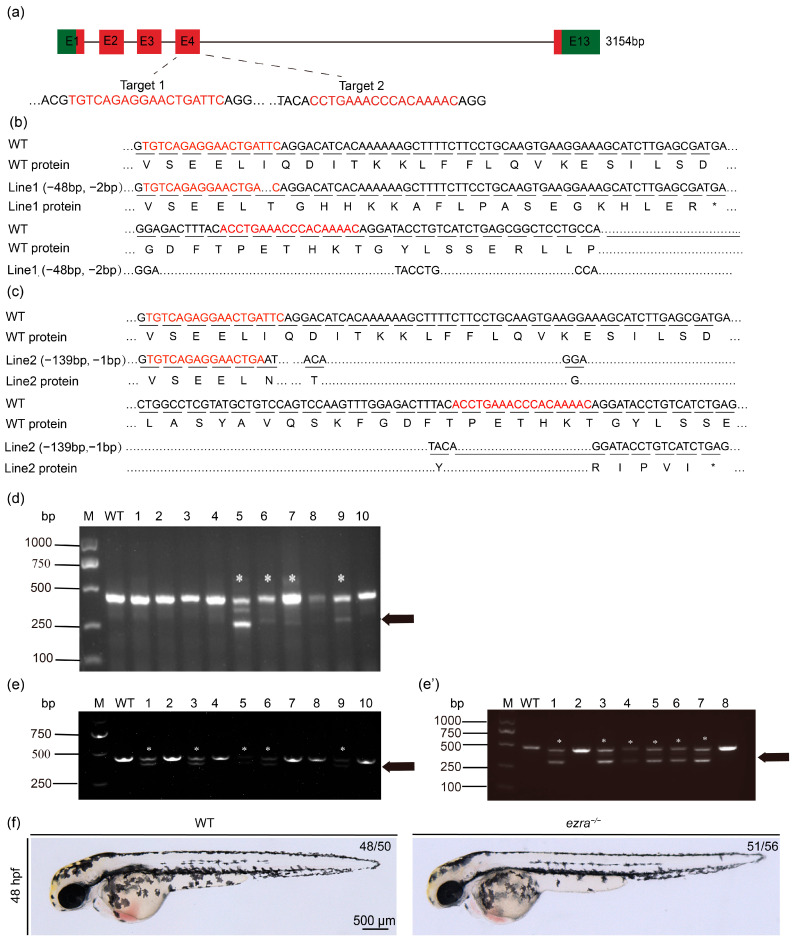
Zebrafish *ezra* gene knockout. (**a**) A schematic of the CRISPR/Cas9 target site in the *ezra* gene. Green boxes indicate 3′-UTR and 5′-UTR, and red boxes represent exons; the target sequence is highlighted in red. (**b**,**c**) The genomic DNA and amino acid sequences of two independent mutant alleles. A 50 bp deletion was identified in the *ezra* mutant line 1 shown in (**b**), and a 140 bp deletion was identified in the *ezra* mutant line 2 shown in (**c**). Deleted bases are indicated by black dotted lines, the target sequence is highlighted in red, and the premature stop codon is denoted by an asterisk (*). (**d**–**e′**) The genotyping results for *ezra*^line2^ F0 adult fish, *ezra*^line1^ F1 adult fish (**e**), and *ezra*^line2^ F1 adult fish (**e′**). Identified mutant adult fish are marked by asterisks (*), M: DNA marker; black arrowheads indicate mutant bands. (**f**) *ezra*^−/−^ exhibit normal development at 48 hpf; the fraction in the upper right corner of panel (**f**) indicates the number of embryos exhibiting the displayed phenotype relative to the total number of embryos examined.

To investigate the role of *ezrin*, the CRISPR/Cas9 gene editing technique was employed to generate an *ezra* knockout mutant line by targeting the fourth exon of *ezra* (Figure 2a). After screening and genotyping, two independent *ezra* mutant lines were identified: mutant line 1 harbored a 50 bp deletion (−48 bp, −2 bp; Figure S1a), and mutant line 2 harbored a 140 bp deletion (−139 bp, −1 bp; Figure S1b). Both mutants resulted in a frameshift in protein translation. Amino acid sequence analysis showed that in *ezra* mutant line 1, the sequence was disrupted from position 94, resulting in a premature stop codon at amino acid 113 (Figure 2b). Similarly, mutant line 2 exhibited a frameshift starting at position 94, leading to premature translation termination at position 150 (Figure 2c). As protein translation terminated prematurely in both lines, the full-length EZRA protein was not produced, confirming that *ezra* function was lost. Heterozygous (Figure 2d–e′) and homozygous (Figure S1c,c’) individuals of the two mutant lines were identified by genotyping. The RT-qPCR experiment results revealed that in *ezra* homozygous mutants (*ezra*^−/−^), *ezra* mRNA was significantly downregulated, whereas *ezrb* mRNA was upregulated, compared to the WT (Figure S1d). Morphological observations further revealed that no significant developmental abnormalities were observed in *ezra*^−/−^ when compared to the WT (Figure 2f).

### 3.3. ezrin Gene Deficiency Leads to Embryonic Cardiac Edema

**Figure 3:**
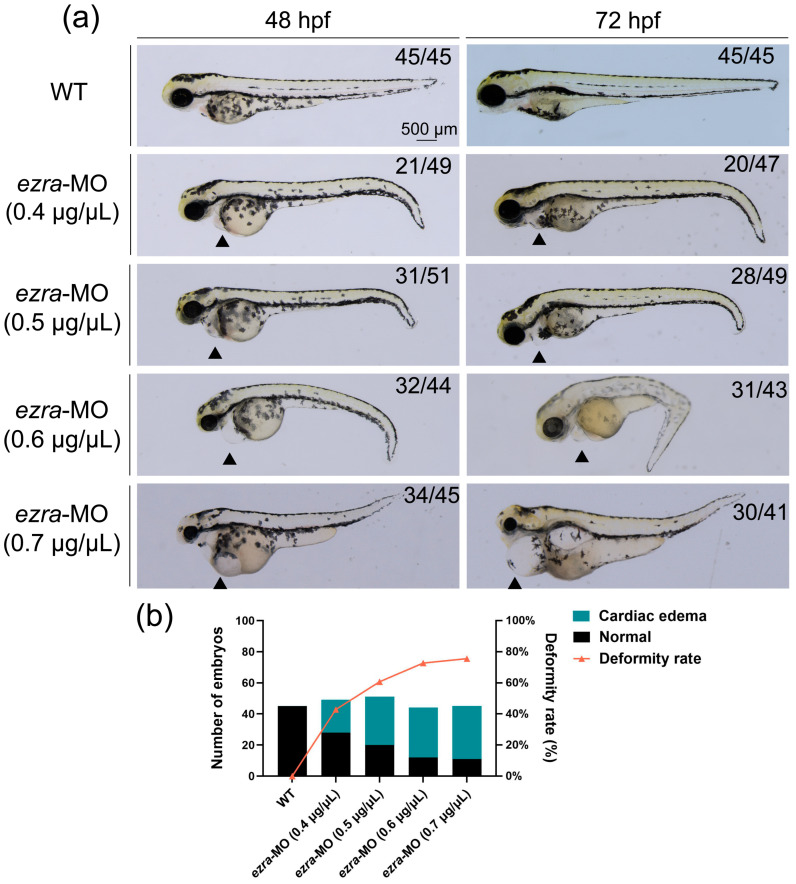
Developmental malformations in zebrafish groups injected with *ezra*–MO. (**a**) Morphological images of embryos injected with different concentrations of *ezra*–MO at 48 hpf and 72 hpf; the fraction in the upper right corner of panels (**a**,**b**) indicates the number of embryos exhibiting the displayed phenotype relative to the total number of embryos examined. (**b**) The statistical results of pericardial edema incidence in *ezra*–MO injection groups at different concentrations are shown. Scale bar: 500 μm; black triangles indicate areas of pericardial edema.

**Figure 4:**
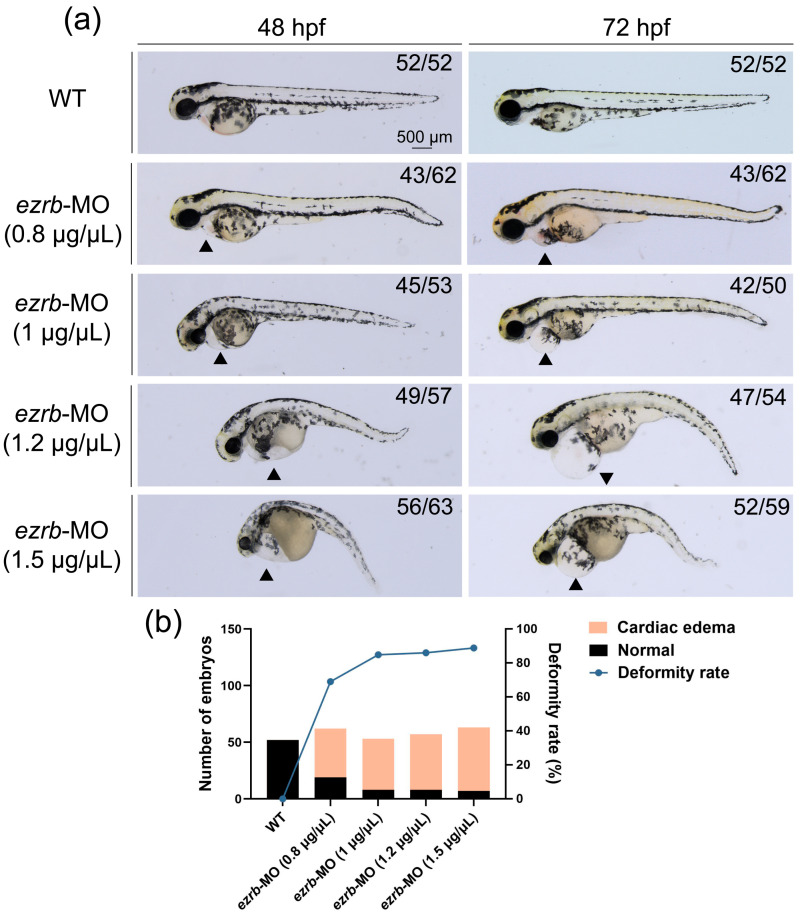
Developmental malformations in zebrafish groups injected with *ezrb*–MO. (**a**) Morphological images of embryos injected with different concentrations of *ezra*–MO at 48 hpf and 72 hpf; the fraction in the upper right corner of panel (**a**) indicates the number of embryos exhibiting the displayed phenotype relative to the total number of embryos examined. (**b**) The statistical results of pericardial edema incidence in *ezra*–MO injection groups at different concentrations are shown. Scale bar: 500 μm; black triangular arrows indicate areas of pericardial edema.

Given the upregulation of *ezrb* in *ezra*^−/−^ embryos, it is probable that *ezrb* functions as a compensatory mechanism in response to *ezra* deficiency, suggesting potential functional redundancy between the two paralogs. To further investigate the function of *ezrin* in development, translation-blocking morpholino oligonucleotides (MOs) targeting the start codons of *ezra* and *ezrb* were synthesized by Gene Tools and used for gene knockdown experiments. To check the optimal injection concentration of *ezra*–MO, four concentrations (0.4, 0.5, 0.6, and 0.7 μg/μL) were micro-injected into one-cell-stage WT embryos. At 48 hpf, a pericardial edema phenotype was observed in *ezra*–MO-injected embryos, which became more pronounced at 72 hpf and increased in severity with higher doses (Figure 3a). A statistical analysis of the malformation rate (Figure 3b) revealed that 0.6 μg/μL induced a relatively stable phenotypic change; consequently, this concentration was selected as the optimal dose. Similarly, four concentrations (0.8, 1.0, 1.2, and 1.5 μg/μL) were evaluated for *ezrb*–MO. Phenotypic analysis revealed pericardial edema at 48 hpf, recapitulating the *ezra*–MO phenotype, with increased severity observed at 72 hpf. Specifically, mild trunk curvature and pericardial edema were observed in the 0.8 μg/μL group, whereas severe embryonic malformations were induced in the 1.5 μg/μL group (Figure 4a). Following statistical analysis, 1 μg/μL was established as the optimal injection concentration for *ezrb*–MO (Figure 4b).

**Figure 5:**
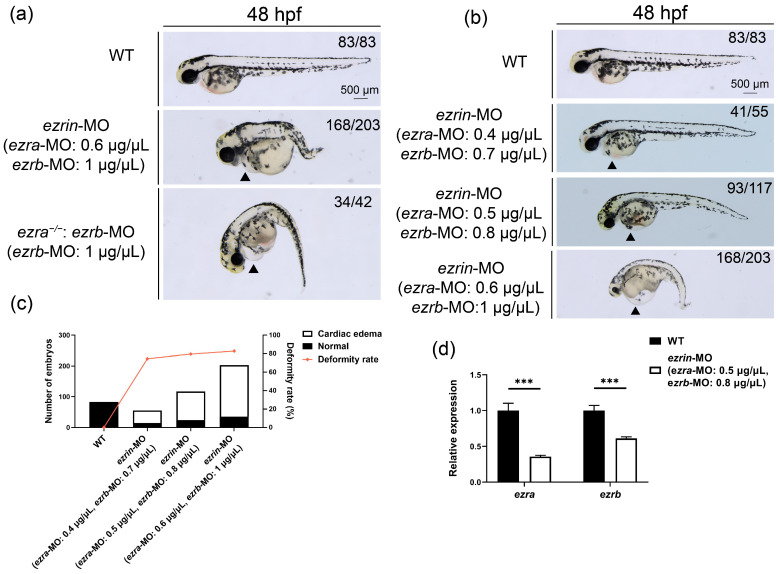
Developmental abnormalities in the zebrafish *ezrin*–MO embryos and the *ezra*^−/−^:*ezrb*–MO embryos. (**a**) Morphological images of the *ezrin*–MO embryos and the *ezra*^−/−^:*ezrb*–MO embryos at 48 hpf. (**b**,**c**) Morphological images (**b**) and statistical results of pericardial edema incidence (**c**) in *ezrin*–MO embryos injected with different concentrations at 48 hpf. The fraction in the upper right corners of panels (**a**,**b**) indicates the number of embryos exhibiting the displayed phenotype relative to the total number of embryos examined. (**d**) The mRNA expression levels of *ezra* and *ezrb* in *ezrin*–MO embryos at 48 hpf. Scale bar: 500 μm; black triangle indicates areas of pericardial edema; *t*-test, ***: *p* < 0.001. For RT-qPCR experiments, three biological replicates were prepared for both WT and *ezrin*–MO embryos, with each replicate containing 50 embryos at 48 hpf.

Based on the optimal concentrations established in the single-gene knockdown experiments, *ezra*–MO and *ezrb*–MO were co-injected into WT embryos, and *ezrb*–MO was injected into *ezra*^−/−^ embryos. At 48 hpf, embryos subjected to both treatments displayed trunk curvature and pericardial edema, with phenotypic severity exceeding that observed in single-knockdown embryos (Figure 5a), suggesting that this exacerbation was likely attributable to excessively high initial co-injection doses. To optimize co-injection conditions, a series of gradient concentrations of the *ezra*–MO and *ezrb*–MO mixture was evaluated. The results demonstrated that the co-injection of *ezra*–MO (0.5 μg/μL) and *ezrb*–MO (0.8 μg/μL) effectively induced pericardial edema while ensuring stable phenotypic penetrance (Figure 5b,c and Figure S2) (hereafter referred to as *ezrin*–MO, instead of a combination of *ezra*–MO [0.5 μg/μL] and *ezrb*–MO [0.8 μg/μL], unless otherwise specifically indicated). The RT-qPCR experiment results confirmed that the co-injection of *ezra*–MO and *ezrb*–MO significantly reduced *ezra* and *ezrb* mRNA levels, demonstrating that MOs effectively suppressed both genes (Figure 5d).

### 3.4. Effects of ezrin Gene Deletion on Cardiac Development

**Figure 6:**
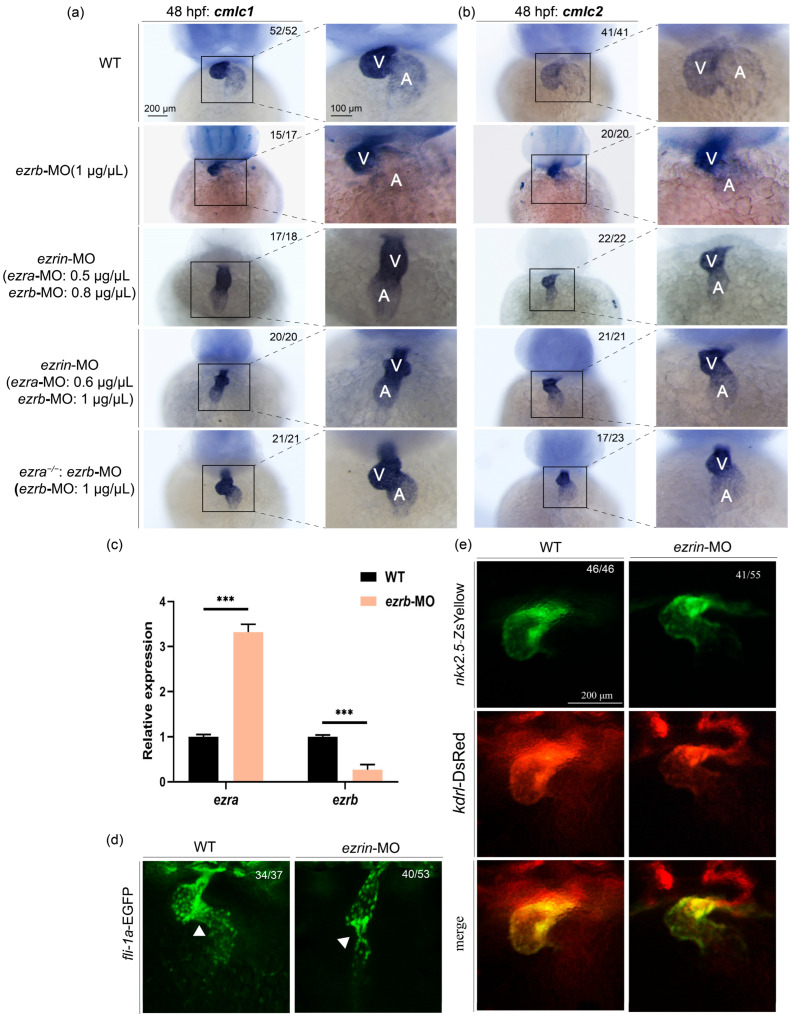
Effects of simultaneous knockdown of *ezra* and *ezrb* genes on zebrafish cardiac development. (**a**,**b**) Ventral view of zebrafish embryos. WISH experiment was performed using *cmlc1* (**a**) and *cmlc2* (**b**) probes to investigate heart development in *ezrb*–MO embryos, *ezrin*–MO embryos, and *ezra*^−/−^: *ezrb*–MO embryos at 48 hpf. Right panels show magnifications of heart region. V: ventricle; A: atrium; scale bar: 200 μm; scale bar for magnified heart region: 100 μm. (**c**) mRNA expression levels of *ezra* and *ezrb* in *ezrb*–MO knockdown embryos. For RT-qPCR experiment, three independent biological replicates were prepared for both WT and *ezrb*–MO embryos, with each replicate consisting of 50 embryos at 48 hpf, *t*-test, ***: *p*< 0.001. (**d**,**e**) Expression of *fli* (**d**), *nkx2.5*, *kdrl* (**e**) in 48 hpf *ezrin*–MO embryos. Scale bar: 200 μm. White triangle indicate atrioventricular valve. Fractions in upper right corners of (**a**,**b**,**d**,**e**) indicate number of embryos with displayed phenotype out of total examined.

To investigate the impact of *ezrin* on heart development in zebrafish, WISH experiment was conducted using antisense RNA probes targeting the cardiac-specific marker genes *cmlc1* and *cmlc2*. The WISH experimental results revealed that, compared to the control, hearts in *ezrb*–MO, *ezrin*–MO, and *ezra*^−/−^:*ezrb*–MO embryos were visibly reduced in size at 48 hpf, with the reduction being more pronounced in double-mutant embryos than in the single-knockdown embryos (Figure 6a,b). These results indicated early heart development in zebrafish embryos was affected by the complete loss of *ezrin* and that functional redundancy may exist between *ezra* and *ezrb*. To investigate this, RT-qPCR was performed on *ezrb*–MO embryos to quantify the mRNA expression of *ezra* and *ezrb*. The RT-qPCR experiment results showed that *ezrb* expression was decreased, whereas *ezra* expression was markedly upregulated. These findings provided preliminary evidence of functional redundancy between *ezra* and *ezrb* (Figure 6c). Furthermore, *ezrin*–MO embryos exhibited a phenotype identical to that of the *ezra*^−/−^:*ezrb*–MO embryos. Consequently, subsequent experiments utilized the double-knockdown embryos. To further characterize this phenotype, *ezra*–MO and *ezrb*–MO were co-injected into transgenic lines expressing green fluorescence in the heart (TL [*nkx2.5*:ZsYellow]), red fluorescence in endothelial cells (Tg [*kdrl*:DsRed]), and green fluorescence in vascular endothelial cells (Tg [*fli-1a*:EGFP]). Subsequent observations revealed that *ezrin*–MO embryos exhibited reduced cardiac chambers and abnormal atrioventricular valve development (Figure 6d,e).

**Figure 7:**
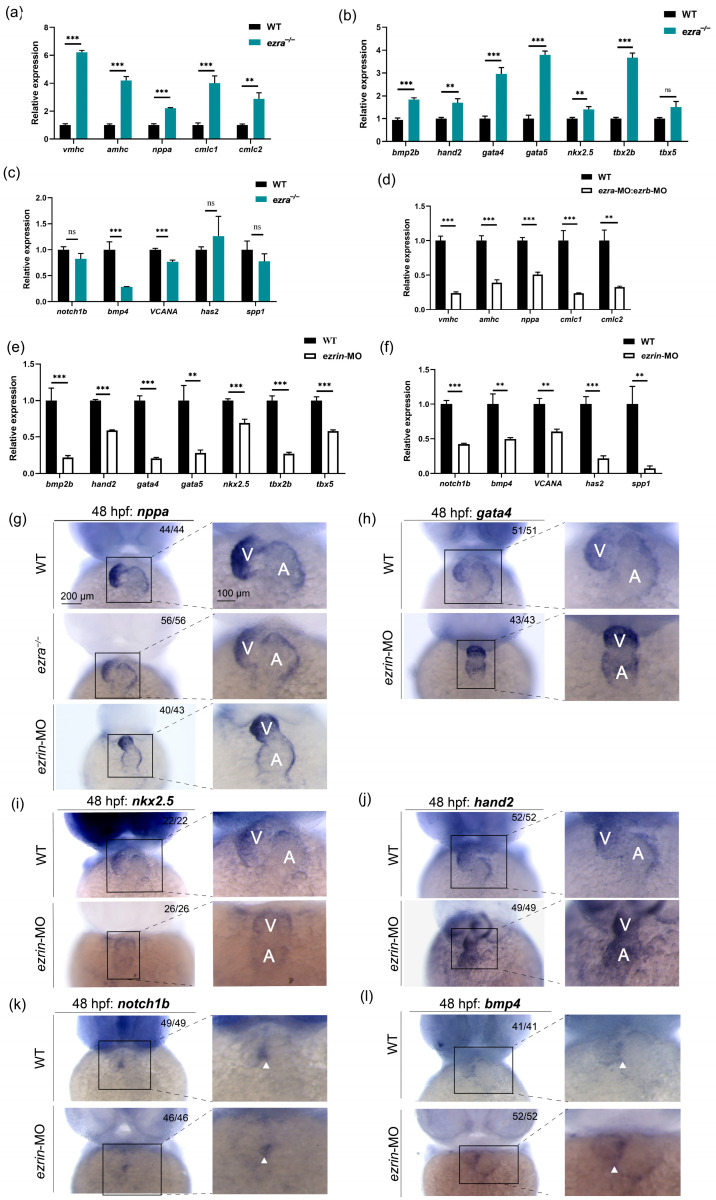
Abnormalities in cardiac and atrioventricular valve development in *ezrin*–MO embryos. (**a**–**f**) Expression levels of cardiac marker genes (**a**,**d**), cardiac developmental regulatory genes (**b**,**e**), and atrioventricular valve marker genes (**c**,**f**) in *ezra*^−/−^ and *ezrin*–MO embryos at 48 hpf; *t*-test, ns: no significance**,** **: *p* < 0.01, ***: *p* < 0.001. For RT-qPCR experiment, three biological replicates were prepared for both WT and *ezrin*–MO embryos, with each replicate containing 50 embryos (48 hpf). (**g**–**i**) Expression of *nppa* (**g**), *gata4* (**h**), *nkx2.5* (**i**), *hand2* (**j**), *notch1b* (**k**), and *bmp4* (**l**) in 48 hpf embryos; right panels show magnified views of heart region; V: ventricle; A: atrium; scale bar: 200 μm; scale bar for magnified heart region: 100 μm, the white triangle indicates the atrioventricular valve region. Fractions in upper right corners of panels (**g**–**l**) indicate number of embryos exhibiting displayed phenotype relative to total number of embryos examined.

To further investigate the effects of *ezrin* deficiency on cardiac development, the expression of relevant genes was analyzed by RT-qPCR experiment as follows. (1) Cardiac marker genes: The ventricular marker *vmhc*, the atrial marker *amhc*, the cardiac myosin markers *cmlc1/2* (facilitating cardiac contraction), and *nppa* (which negatively regulates cardiac hypertrophy and fibrosis). (2) Cardiac development regulatory genes: *tbx2b*, which inhibits chamber differentiation and proliferation [65]; *tbx5*, which guides the directed migration of cardiac progenitor cells [66]; *bmp2b*, a core transcription factor required for chamber contraction and morphogenesis; *hand2*, which promotes cardiomyocyte proliferation; *gata4*, which is essential for outflow tract formation [67]; *gata5*, a regulator of cardiomyocyte precursor development and cardiac primordium formation; and *nkx2.5*, an early cardiac lineage marker [68]. (3) Atrioventricular valve development marker genes: The atrioventricular canal endothelial marker *notch1b*, the atrioventricular canal myocardial markers *bmp4* and *VCANA* (*versican*) [69,70], and the endocardial cushion markers *has2* and *spp1*. The RT-qPCR experimental results showed that the expression of cardiac marker and development-related genes was upregulated in *ezra*^−/−^ (Figure 7a,b), whereas the expression of the atrioventricular valve markers *bmp4* and *VCANA* was downregulated (Figure 7c). In the *ezrin*–MO embryos, the expression levels of all aforementioned genes were significantly suppressed (Figure 7d–f). To validate the RT-qPCR experimental findings, WISH was employed to examine the expression of *nppa*, *gata4*, *nkx2.5*, *hand2*, *bmp4*, and *notch1b*. The WISH experimental results demonstrated that the spatial pattern of *nppa* in *ezra*^−/−^ and WT embryos was similar, whereas in the *ezrin*–MO embryos, the expression of *nppa*, *gata4*, *hand2*, *nkx2.5*, *bmp4*, and *notch1b* was significantly downregulated (Figure 7g–i), consistent with the RT-qPCR experimental results. These observations suggest that the *ezra* and *ezrb* genes may function synergistically during cardiac development and that the loss of *ezrin* function may result in cardiac and atrioventricular valve development defects.

### 3.5. Effects of ezrin Gene Deletion on Zebrafish Transcriptome

**Figure 8:**
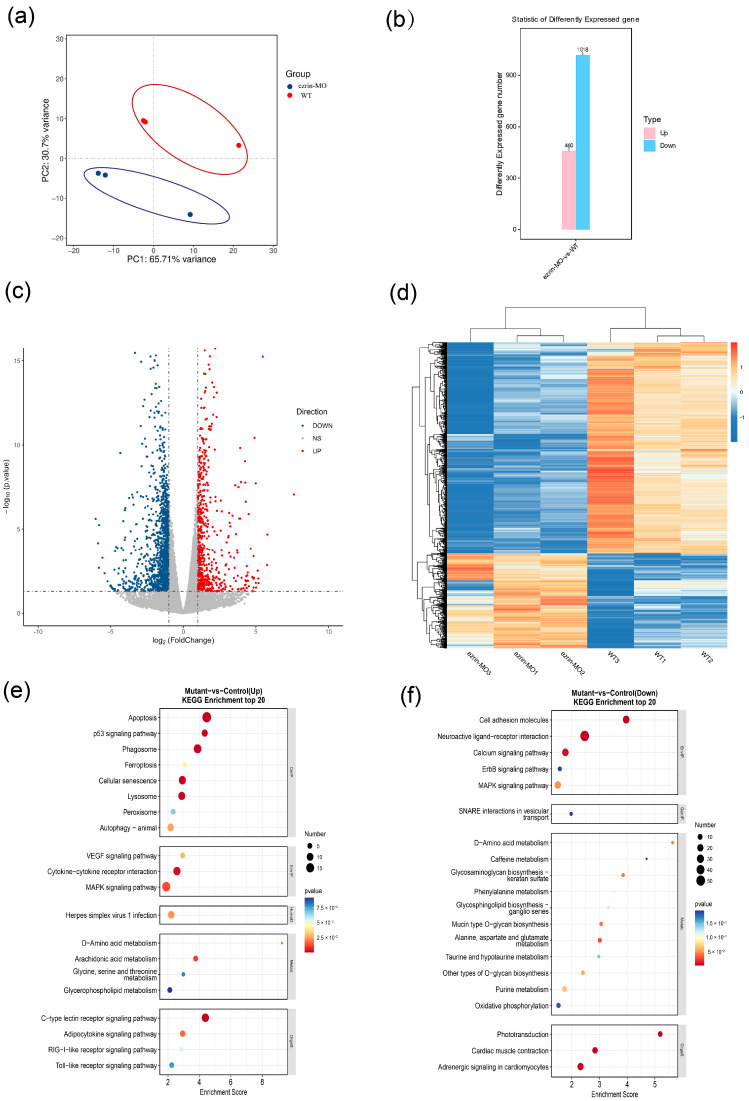
An analysis of DEGs between the *ezrin*–MO embryos and the WT at 48 hpf. (**a**) PCA was performed on WT and *ezrin*–MO embryo samples. (**b**) The number of DEGs between WT and *ezrin*–MO embryo samples is shown, with red indicating upregulated genes and blue indicating downregulated genes. (**c**) A volcano plot visualized the distribution of DEGs in WT and *ezrin*–MO embryo samples. The dashed line represents the fold change threshold. (**d**) A clustering heatmap of WT and *ezrin*–MO embryo samples. (**e**,**f**) The KEGG analysis of upregulated (**e**) and downregulated (**f**) DEGs shows significantly enriched pathways, with the x-axis representing the enrichment score and the y-axis representing the enriched pathway names.

To investigate the molecular mechanisms underlying the cardiac developmental defects associated with *ezrin* deficiency in zebrafish, high-throughput RNA sequencing was conducted on 48 hpf WT and *ezrin*–MO embryos, with three biological replicates in each group. The quality of the sequencing data met the standards recommended by the ENCODE Consortium. Principal component analysis (PCA) was employed to evaluate sample heterogeneity. Analysis revealed a clear segregation between the experimental (*ezrin*–MO) and control (WT) groups, with high intragroup reproducibility (Figure 8a). Applying a threshold of |log_2_FoldChange| ≥ 1 and adjusted *p* ≤ 0.05, a total of 1478 DEGs were identified, comprising 460 upregulated and 1018 downregulated transcripts (Figure 8b). A volcano plot visualized the distribution of DEGs (Figure 8c), while a hierarchical clustering heatmap corroborated the significant divergence between samples (Figure 8d). KEGG pathway enrichment analysis displayed that upregulated DEGs were enriched in the p53 signaling pathway, apoptosis, and the MAPK signaling pathway (Figure 8e), whereas downregulated DEGs were primarily enriched in the cardiac muscle contraction pathway, adrenergic signaling in cardiomyocytes, and the calcium signaling pathway (Figure 8f).

### 3.6. ezrin Gene Deficiency Affects Cardiac Contraction and Activates Apoptotic Pathways

**Figure 9:**
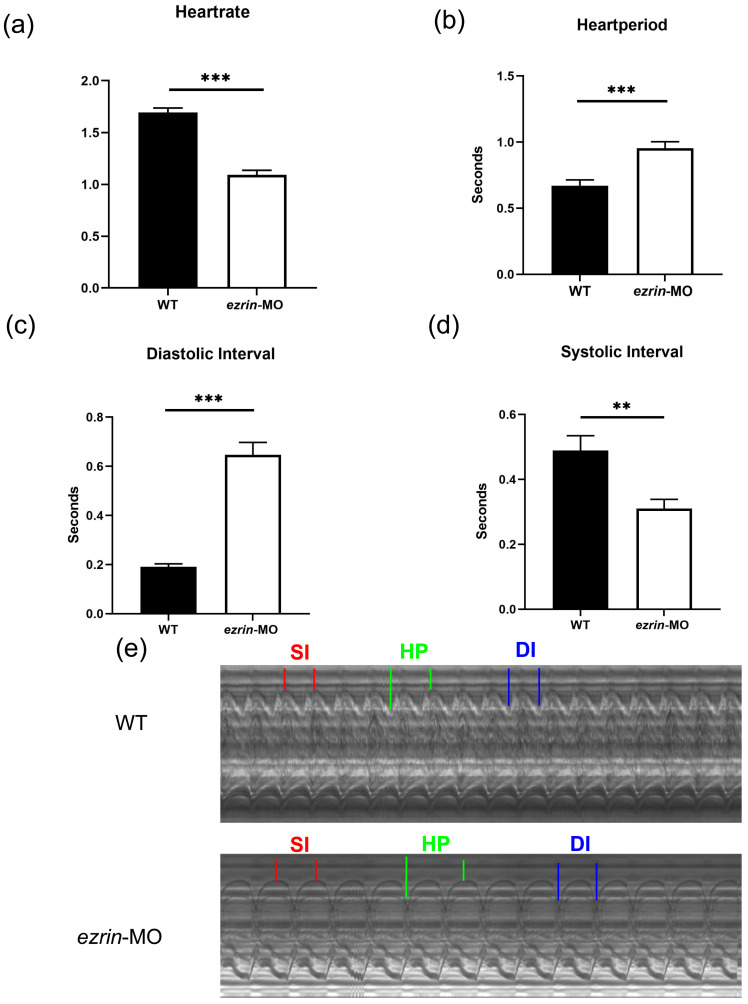
Double knockdown of *ezrin*–MO affects embryonic cardiac contractile function. HR (**a**), HP (**b**), DI (**c**), and SI (**d**) in *ezrin*–MO embryos at 48 hpf. (**e**) M-mode traces from representative heartbeat videos of WT and *ezrin*–MO embryos; *t*-test, **: *p* < 0.01, ***: *p* < 0.001.

Given the enrichment in downregulated genes in cardiac contraction-related signaling pathways in the *ezrin*–MO embryos, the effects of *ezrin* deficiency on cardiac contractile function were further examined. The dynamic imaging of the ventricular region in 48 hpf embryos was acquired at 150 frames s^−1^ utilizing an EM-CCD. Each video spanned 10 s, with 20 embryos per group randomly chosen for imaging using a 10× objective. All video data were converted to M-mode tracings and processed using semi-automated heartbeat analysis software (v3.4.0.0) to quantify HR, HP, DI, and SI. The results are shown in Figure 9. Compared with the control group, *ezrin*–MO embryos displayed a significantly reduced heart rate (Figure 9a), concomitant with markedly prolonged HP (Figure 9b), DI (Figure 9c), and SI (Figure 9d). Representative M-mode images further demonstrated the altered cardiac cycle parameters (Figure 9e). These findings suggested that the simultaneous suppression of the *ezra* and *ezrb* genes impairs early cardiac contractile function, leading to bradycardia.

**Figure 10:**
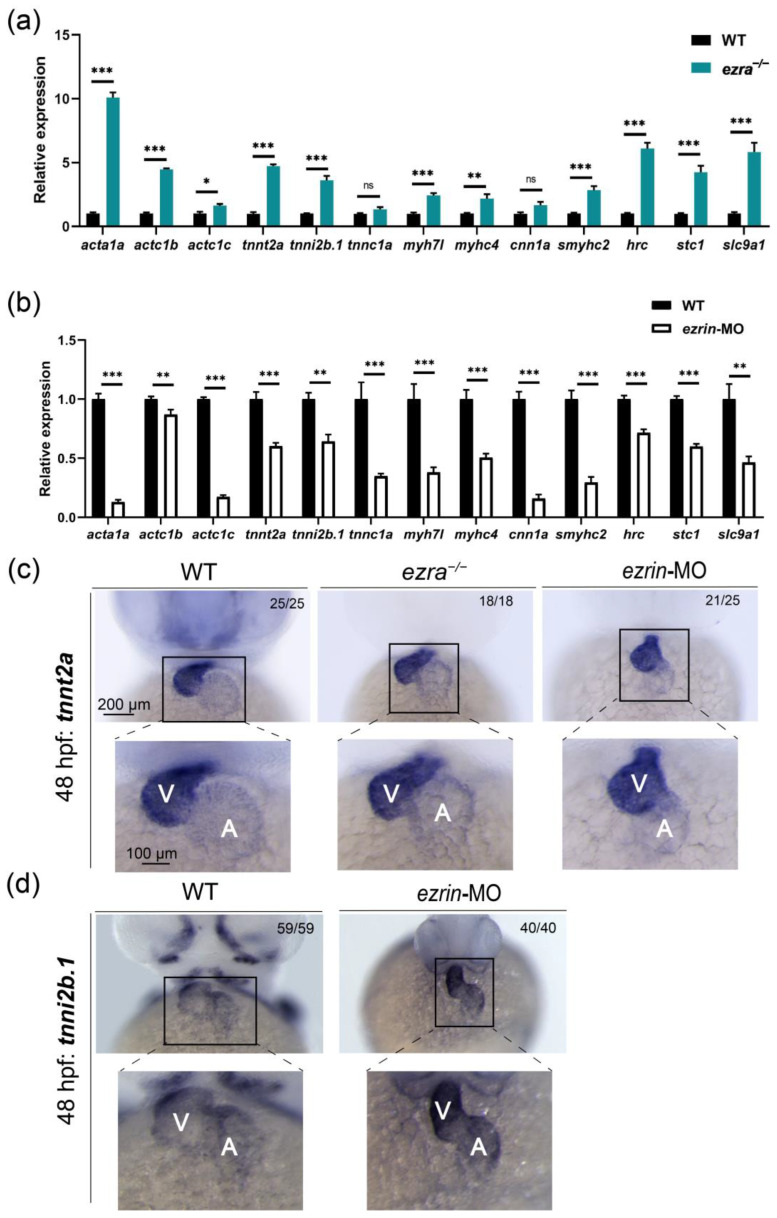
Simultaneous knockdown of *ezra* and *ezrb* leads to aberrant expression of genes associated with myocardial contraction. (**a**,**b**) Expression levels of myocardial contraction-related genes in *ezra*^−/−^ (**a**) and *ezrin*–MO embryos (**b**) at 48 hpf. *t*-test, ns: no significance**,** *: *p* < 0.05, **: *p* < 0.01, ***: *p* < 0.001. For RT-qPCR experiment, three independent biological replicates were prepared for WT, *ezra*^−/−^, and *ezrin*–MO embryos, with each replicate consisting of 50 embryos at 48 hpf. (**c**,**d**) Expression of *tnnt2a* (**c**) and *tnni2b.1* (**d**) in embryos at 48 hpf was examined using WISH experiment. Right panels show magnified views of heart region. V: ventricle; A: atrium; scale bar: 200 μm; scale bar for magnified heart region: 100 μm; fraction in upper right corners of panels (**c**,**d**) indicates number of embryos exhibiting displayed phenotype relative to total number of embryos examined.

To investigate the molecular mechanisms underlying the observed contractile dysfunction, RT-qPCR experiment was employed to examine the expression of genes associated with myocardial contraction. The genes analyzed included *acta1a*, a regulator of cardiac myocyte differentiation; *actc1b*, which participates in troponin complex assembly; *actc1c*, which regulates skeletal muscle fiber formation; *tnnt2a* [71], which is required in the assembly of the myosin–troponin complex and is closely associated with the onset of cardiac diseases; *tnni2b.1* and *tnnc1a*, which are involved in cardiac contraction and ventricular development; *myh7l*, which contributes to ventricular development and inhibits cardiac hypertrophy; *myhc4*, which is involved in actin filament binding; *cnn1a*, which mediates actin cytoskeletal organization; *smyhc2*, a slow-twitch marker gene involved in slow skeletal muscle fiber contraction; *slc9a1*, a sodium–potassium ion transmembrane transporter; *hrc*, which possesses calcium-binding activity; and *stc1*, which is involved in intracellular calcium homeostasis. The RT-qPCR experimental results revealed that, compared to WT embryos, the expression of these cardiac contraction-associated genes was significantly upregulated in *ezra*^−/−^ embryos (Figure 10a), whereas their expression was uniformly and markedly suppressed in *ezrin*–MO embryos (Figure 10b).

WISH were further employed to validate the expression patterns of *tnnt2a* and *tnni2b.1*. The results demonstrated that *tnnt2a* expression in *ezra*^−/−^ embryos was comparable to that in the WT. Conversely, in the *ezrin*–MO embryos, the expression of *tnnt2a* and *tnni2b.1* was diminished (Figure 10c,d). Collectively, these findings suggest that *ezrin* may play a crucial regulatory role in cardiac and atrioventricular valve morphogenesis and the maintenance of normal myocardial contraction.

**Figure 11:**
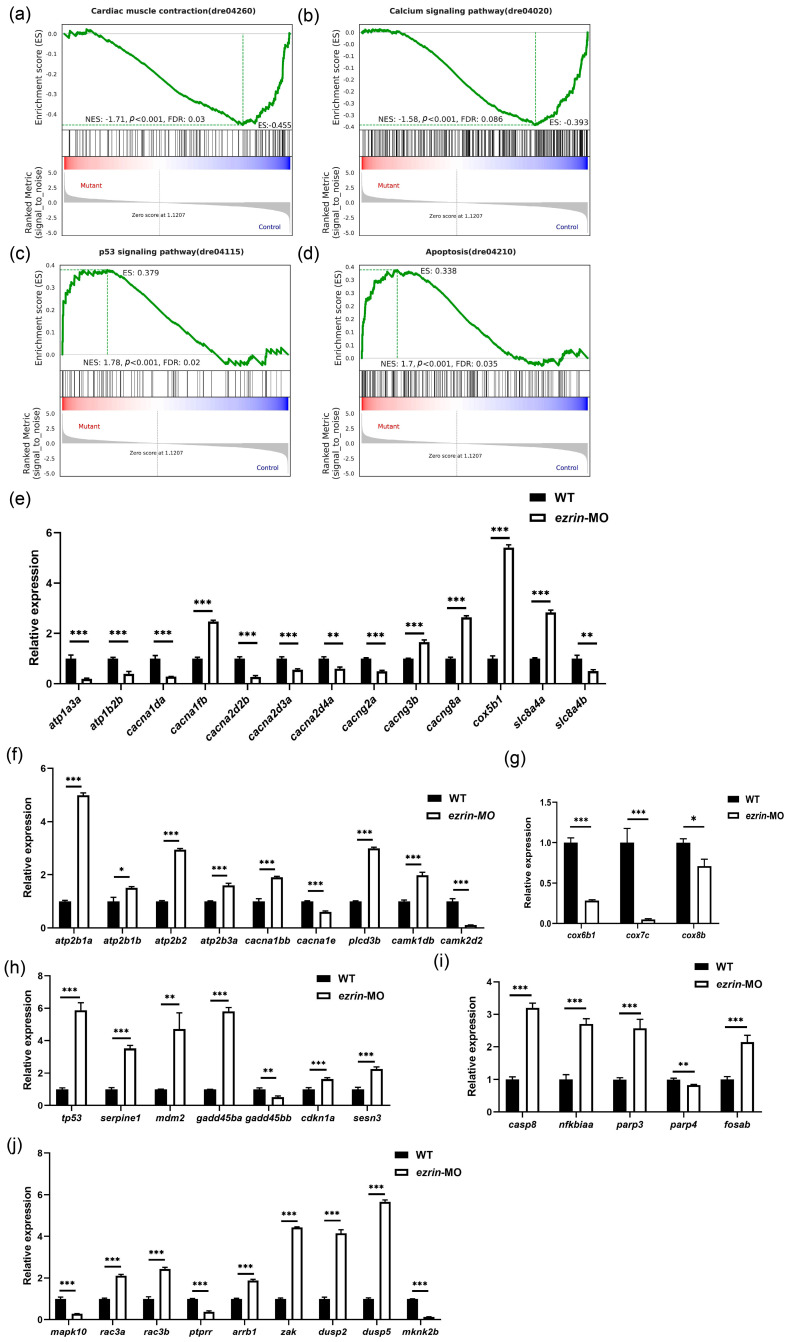
*ezrin* is required for myocardial contractile function and apoptosis signaling pathways. (**a**–**d**) GSEA enrichment plots for the cardiac muscle contraction pathway (**a**), calcium signaling pathway (**b**), p53 signaling pathway (**c**), and apoptosis signaling pathway (**d**). The left vertical axis represents the enrichment score and signal-to-noise ratio. The green curve indicates the enrichment profile, and the black lines in the middle represent the positions of individual genes. The green dashed line denotes the peak ES, the grey line represents ES = 0, and the color bar indicates the strength of gene–phenotype association, with red representing genes highly expressed in the Mutant and blue representing those highly expressed in the Control. NES, normalized enrichment score; FDR, false discovery rate; zero score, zero crossing. (**e**–**j**) The expression levels of genes associated with the cardiac muscle contraction pathway (**e**), calcium signaling pathway (**f**), cytochrome C oxidase subunit (**g**), p53 signaling pathway (**h**), apoptosis signaling pathway (**i**), and MAPK signaling pathway in *ezrin*–MO embryos at 48 hpf. *t*-test, *: *p* < 0.05, **: *p* < 0.01, ***: *p* < 0.001. For RT-qPCR experiment analyses, three independent biological replicates were prepared for both WT and *ezrin*–MO embryos, with each replicate consisting of 50 embryos at 48 hpf.

The RNA-seq results revealed that *ezrin* deficiency led to the aberrant expression of genes associated with myocardial contraction and promoted apoptosis, including cardiac muscle contraction, calcium signaling, p53 signaling, and the apoptosis pathway (Figure 11a–d). By integrating KEGG pathway enrichment analysis with phenotypic observations, RT-qPCR experiment was used to validate the expression changes in key genes in the cardiac muscle contraction pathway. Within this pathway, the expression of *atp1a3a*, which is involved in ventricular development, and *atp1b2b*, which is expressed in the zebrafish heart, was significantly downregulated. The voltage-gated calcium channel subunit genes *cacna1da*, *cacna2d2b*, *cacna2d3a*, *cacna2d4a*, and *cacng2a* were significantly downregulated, whereas *cacna1fb*, *cacng3b*, and *cacng8a* were upregulated. Additionally, the mitochondrial cytochrome C oxidase subunit gene *cox5b1* and the sodium–calcium exchanger gene *slc8a4a* were upregulated, whereas *slc8a4b* was downregulated (Figure 11e). These results suggest that *ezrin* deficiency may impair embryonic heart development and contractile function by disrupting calcium homeostasis in cardiomyocytes. A further analysis of genes associated with the calcium signaling pathway and mitochondrial function revealed that the plasma membrane Ca^2+^-ATPase (PMCA) subunit genes *atp2b1a*, *atp2b1b*, *atp2b2*, and *atp2b3a* were significantly upregulated (Figure 11f). The primary function of PMCA is to extrude cytoplasmic Ca^2+^ against the concentration gradient, thereby maintaining intracellular Ca^2+^ homeostasis [72]. The voltage-gated calcium channel gene *cacna1e* was downregulated, whereas *cacna1bb* was upregulated. The calcium signaling downstream component *plcd3b* and the calmodulin-dependent protein kinase *camk1db* were upregulated, whereas *camk2d2* was suppressed. Notably, mitochondrial electron transport chain-related genes *cox6b1*, *cox7c*, and *cox8b* were significantly downregulated (Figure 11g), further suggesting that *ezrin* deficiency may perturb calcium homeostasis and compromise mitochondrial function in cardiomyocytes.

Furthermore, KEGG pathway enrichment analysis revealed that the apoptosis, p53 signaling, and MAPK signaling pathways were activated. Within the p53 signaling pathway, significantly upregulated genes included *tp53*, a core regulator of intrinsic apoptosis; *serpine1*, an inhibitor of plasminogen activation; *mdm2*, an apoptosis inhibitor; *gadd45ba*, a DNA damage-induced pro-apoptotic factor; *cdkn1a*, a cell cycle inhibitor [73]; and *sesn3*, an antioxidant gene, whereas *gadd45bb*, associated with somatic development, was downregulated (Figure 11h). In the apoptosis pathway (Figure 11i), the expression of the extrinsic apoptosis regulator *casp8* [74]; *nfkbiaa*, which is involved in overall cell survival; *parp3*, which participates in various cellular events (including genomic integrity, transcription, differentiation, cellular metabolism, and cell death); and *fosab*, which is involved in zebrafish cardiac tissue regeneration [75], was upregulated. In contrast, *parp4*, which catalyzes the ADP ribosylation of target proteins using NAD+ as a substrate, was downregulated. Within the MAPK signaling pathway, the expression of *mapk10*, a regulator of proliferation, differentiation, transcriptional regulation, and development; *ptprr*, a modulator of signal transduction via MAPK activity downregulation, and *mknk2b*, which possesses calcium/calmodulin-dependent protein kinase activity, was significantly downregulated. Conversely, upregulation was detected for *rac3a*, which possesses GTPase activity; *rac3b*, which participates in actin structure organization; *zak*, which implicates signal transduction and protein phosphorylation; *arrb1*, which encodes β-arrestin and replaces the heterotrimeric G protein (Gs) to induce signal transduction via the MAPK pathway; and *dusp2* and *dusp5*, negative regulators of the MAPK signaling cascade (Figure 11j). These findings indicate that *ezrin* deficiency triggers the p53, apoptosis, and MAPK signaling pathways, potentially influencing cell fate determination and cardiac morphogenesis in concert.

## 4. Discussion

Using zebrafish as a model, our results demonstrated that the *ezrin* gene plays a crucial role in early cardiac morphogenesis and the maintenance of myocardial contractile function, revealing functional redundancy between *ezra* and *ezrb*. The WISH and RT-qPCR experiments results revealed that both *ezra* and *ezrb* are expressed maternally and ubiquitously in early developmental stages, suggesting that both genes may function in early embryogenesis. Although *ezra^-/-^* did not exhibit obvious morphological abnormalities, the RT-qPCR experiment results revealed that *ezrb* expression was upregulated in *ezra*^−/−^ embryos. Conversely, *ezra* expression was compensatorily elevated in *ezrb*–MO embryos. However, the simultaneous knockdown of *ezra* and *ezrb* using translation-blocking MOs induced pericardial edema, diminished cardiac chamber size, and atrioventricular valve malformations. Phenotype severity correlated with MO dose and was substantially more severe than that observed in single-knockdown embryos. The mild trunk curvature and overall developmental delay observed in *ezrin*–MO embryos may be attributable to secondary effects arising from pericardial edema and cardiac developmental defects. These findings indicate that *ezra* and *ezrb* exhibit functional redundancy and likely act synergistically in regulating cardiac morphogenesis. The abrogation of both genes may lead to cardiac developmental abnormalities.

As the earliest functional organ to emerge during zebrafish embryogenesis, the heart undergoes complex morphogenetic remodeling regulated by key transcription factors and signaling molecules, including *vmhc*, *nkx2.5*, *gata4/5*, and *nppa* [76]. RT-qPCR experiment was employed to quantify the expression of cardiac marker genes (*vmhc*, *amhc*, *cmlc1/2*, etc.), cardiac developmental regulatory genes (*tbx2b*, *gata4/5*, *nkx2.5*, etc.), and atrioventricular valve marker genes (*bmp4*, *notch1b*, etc.). In *ezra*^−/−^, cardiac markers and developmental regulatory genes were significantly upregulated, suggesting that *ezrb* may be compensatorily activated following the loss of *ezra* to maintain cardiac developmental homeostasis. However, the atrioventricular valve markers *bmp4* and *VCANA* were significantly downregulated in *ezra*^−/−^, implicating that *ezra* may be involved in the myocardial differentiation of the atrioventricular valve. In contrast, in *ezrin*–MO embryos, the expression of all the aforementioned genes was significantly downregulated. This suggests functional redundancy between *ezra* and *ezrb*; the simultaneous loss of both genes abolishes the compensatory mechanism, resulting in abnormal embryonic cardiac development and preliminarily reveals the importance of *ezrin* in maintaining normal cardiac morphology. The spatial expression analysis of genes such as *nppa* and *nkx2.5* by WISH experiment further confirmed the reduced cardiac chamber size and atrioventricular valve developmental defects observed in *ezrin*–MO embryos, providing preliminary support for the hypothesis that *ezrin* may occupy a pivotal position within the cardiac developmental regulatory network.

To elucidate the molecular mechanism responsible for cardiac defects induced by *ezrin* deficiency, RNA-seq was conducted on *ezrin*–MO embryos, subsequently subjected to KEGG pathway enrichment analysis. Upregulated DEGs were enriched in the p53 signaling pathway, apoptosis pathway, and MAPK signaling pathway, whereas downregulated DEGs were predominantly enriched in cardiac muscle contraction and calcium signaling pathways. Concomitantly, cardiac physiology analysis demonstrated bradycardia and a significant prolongation of the heart period, diastolic period, and systolic period in the double knockdown. A panel of genes implicated in cardiac contractile function (including *actc1b*, *tnnt2a*, and *hrc*) was selected for RT-qPCR experiment validation. These contraction-associated genes were significantly upregulated in *ezra*^−/−^ embryos, whereas their expression was suppressed in *ezrin*–MO embryos. WISH experimental result confirmed the diminished expression of *tnnt2a* and *tnnt2b.1* in double-knockdown embryos. Integrated with previously observed phenotypes, such as diminished cardiac chamber size, these findings suggest that *ezrin* deficiency results in not only cardiac developmental malformations but also impaired myocardial contractility. This phenomenon may be mediated by a dual mechanism: first, the induction of cardiomyocyte apoptosis through pro-apoptosis signaling pathways, and second, the disruption of calcium homeostasis within cardiomyocytes via the dysregulation of calcium signaling.

At the level of myocardial contraction regulation, the dysregulation of calcium signaling may disturb calcium homeostasis within cardiomyocytes, thereby impairing myocardial contractility. Specifically, *ezrin* deficiency leads to the downregulation of the ventricular development genes *atp1a3a* and *atp1b2b*, indicating the impairment of normal embryonic heart development. Multiple voltage-gated calcium channel subunit genes (*cacna1da*, *cacna2d2b*, *cacna2d3a*, *cacna2d4a*, and *cacng2a*) [77] and the calcium transporter *slc8a4b* were downregulated, inhibiting calcium ion transmembrane transport and consequently impairing myocardial contractility. The upregulation of certain calcium channel-related genes (*cacna1fb*, *cacng3b*, and *cacng8a*, etc.) and the calcium transporter *slc8a4a* suggested that *ezrin*-deficient embryos attempted to maintain calcium homeostasis by activating other calcium channels; however, this compensatory response was clearly insufficient to rescue cardiac developmental defects. The upregulation of PMCA subunits (*atp2b1a*, *atp2b1b*, *atp2b2*, and *atp2b3a*) suggested that *ezrin* deficiency may activate P-type calcium transporters, leading to the excessive efflux of intracellular Ca^2+^ and consequently disrupting calcium homeostasis within cardiomyocytes. The upregulation of *plcd3b*, a downstream molecule of the calcium signaling pathway, and calmodulin-dependent protein kinase *camk1db* [78] suggested that the IP3-mediated endoplasmic reticulum calcium release pathway may be compensatorily activated to maintain intracellular calcium homeostasis, whereas the downregulation of *camk2d2* [79] may limit the efficacy of this compensatory mechanism. Additionally, the expression of *cox6b1*, *cox7c*, and *cox8b* was significantly downregulated, suggesting potential energy metabolic disturbances in cardiomyocytes. This mitochondrial dysfunction may not only exacerbate the disruption of intracellular Ca^2+^ homeostasis but also impair myocardial contractility. The upregulation of cytochrome oxidase subunit *cox5b1* [80] may represent a limited compensatory response to energy stress.

*tp53*, a central regulator of the p53 signaling pathway, has been implicated in diverse cellular processes, including apoptosis and cell cycle inhibition [81]. The upregulation of *tp53* suggested that *ezrin* deficiency may activate cardiomyocyte apoptosis. *gadd45ba* and *gadd45bb* are growth arrest and DNA damage-induced genes; the former is involved in apoptosis, while the latter is associated with somatic cell differentiation. The upregulation of *gadd45ba* suggested that cardiomyocytes may undergo apoptosis, whereas the downregulation of *gadd45bb* indicated that cardiomyocyte differentiation may be inhibited. The upregulation of *Cdkn1a*, a cell cycle inhibitor associated with aortic development, suggested that cardiomyocyte proliferation and aortic development may be impaired. The upregulation of *sesn3* suggested that the organism may enhance its resistance to oxidative stress by reducing intracellular reactive oxygen species. Concurrently, the upregulation of *casp8* indicated the activation of the extrinsic apoptosis pathway [82]. *Nfkbiaa*, a gene involved in overall cell survival and serving as a key indicator of development, was upregulated, suggesting that it may affect the normal expression of anti-apoptotic genes by inhibiting NF-κB. *Parp3* participates in various post-translational modifications to promote, control, or regulate numerous cellular events, including genomic integrity, transcription, differentiation, cellular metabolism, and cell death; its upregulation may indicate impaired DNA damage repair. The upregulation of *fosab* suggested that the organism attempted to compensate for cardiac developmental abnormalities caused by *ezrin* deficiency by regulating myocardial tissue regeneration. The MAPK signaling pathway may influence cardiac development through multiple mechanisms. For instance, the downregulation of *mapk10* [83]—which participates in regulating proliferation and differentiation—and *mknk2b*, which possesses calcium/calmodulin-dependent protein kinase activity, suggested that the proliferation and differentiation of cardiomyocytes may be inhibited. The upregulation of *zak* and *arrb1*, which are involved in signal transduction, suggested that the JNK and p38 MAPK signaling pathways were activated. *dusp2* and *dusp5*, as negative regulators in the MAPK signaling pathway, may influence the expression of the regenerative repair gene *fosab* by regulating the dephosphorylation of *mapk10*. Based on the synergistic changes in the aforementioned signaling pathways, it is hypothesized that *ezrin* deficiency may lead to cardiac morphological abnormalities and contractile dysfunction by activating pro-apoptotic signaling cascades and inhibiting cardiomyocyte proliferation and differentiation.

**Figure 12:**
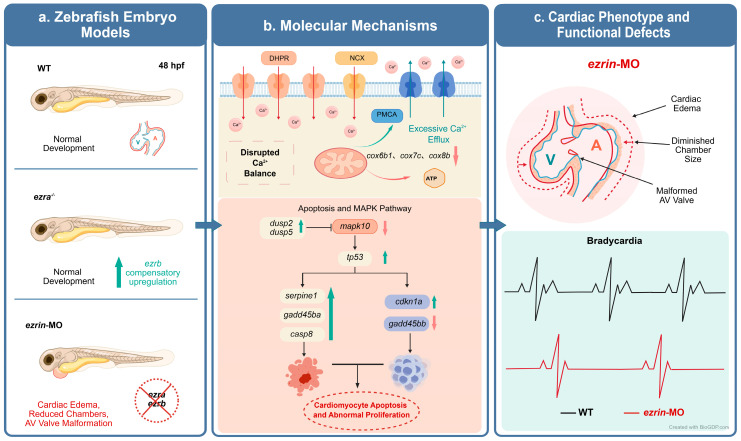
The role of *ezrin* in cardiac morphogenesis and functional maintenance. (**a**) The zebrafish model was established in this study. (**b**) The molecular mechanism by which *ezrin* depletion affects cardiac development. (**c**) Embryonic phenotypes resulting from *ezrin* depletion. Green arrowheads indicate upregulation or activation; red arrowheads indicate downregulation or inhibition; red crosses indicate blocked gene translation. V: ventricle; A: atrium. Created with BioGDP.com [84].

In summary, this study provides preliminary evidence that the *ezrin* gene plays a crucial role in zebrafish cardiac development and function and that functional redundancy exists between *ezra* and *ezrb*. Furthermore, by integrating transcriptomic profiling, cardiac physiological function analysis, and gene expression analysis, our results showed that *ezrin* deficiency may synergistically cause cardiac developmental defects and abnormal myocardial contractile function through the activation of apoptosis, dysregulation of calcium homeostasis within cardiomyocytes, and perturbation of the MAPK signaling pathway mediating cardiomyocyte proliferation and differentiation. These findings provide a new perspective on the role of the *ezrin* gene in cardiac development and offer a new framework for understanding the molecular basis of vertebrate heart development (Figure 12).

## 5. Conclusions

This study provides preliminary evidence that the *ezrin* gene is essential for cardiac morphogenesis and functional maintenance, with functional redundancy between *ezra* and *ezrb*. *ezrin* deficiency may lead to cardiac developmental defects and contractile dysfunction by activating apoptosis, disrupting calcium homeostasis, and perturbing the MAPK signaling pathway mediating cardiomyocyte proliferation and differentiation.

## Data Availability

The datasets utilized and/or examined in this investigation are accessible from the corresponding author upon a reasonable request. The RNA sequencing data generated in this study were archived in the NCBI Sequence Read Archive (SRA) under the accession number PRJNA1459656.

## Supplementary Materials

The following supporting information can be downloaded at https://www.mdpi.com/article/10.3390/cells15121046/s1. Figure S1: *ezra* gene knockout; Figure S2: Morphological images of *ezrin*–MO embryos injected with different concentrations at 72 hpf; Table S1: Primers of RT-qPCR experiment; Table S2: WISH experiment probe primers.

## Abbreviations

The following abbreviations are used in this manuscript:

hpf	hours post-fertilization
dpf	days post-fertilization
mRNA	messenger RNA
PAM	protospacer adjacent motif
sgRNA	single-guide RNA
FHF	first heart field
SHF	second heart field
ECM	extracellular matrix
bp	base pair
NCBI	National Center for Biotechnology Information
BCIP	5-bromo-4-chloro-3-indolyl phosphate p-toluidine salt
AVC	atrioventricular canal
OFT	outflow tract
Edcs	endocardial cells
EMT	epithelial–mesenchymal transition
BCR	B-cell receptor
NBT	Nitrotetrazolium Blue chloride
