# Supplementary material for: The ezrin Gene Regulates Early Cardiac Morphogenesis and Contractile Function in Zebrafish Through the Coordinated Regulation of Apoptosis, Calcium Homeostasis, and the MAPK Signaling Pathway
Source: Cells. 2026 Jun 7;15(12):1046. doi: 10.3390/cells15121046 (PMC13297099; doi:10.3390/cells15121046)
Supplement: Supplementary file 1 [file cells-15-01046-s001.zip › cells-4322470-supplementary.pdf]

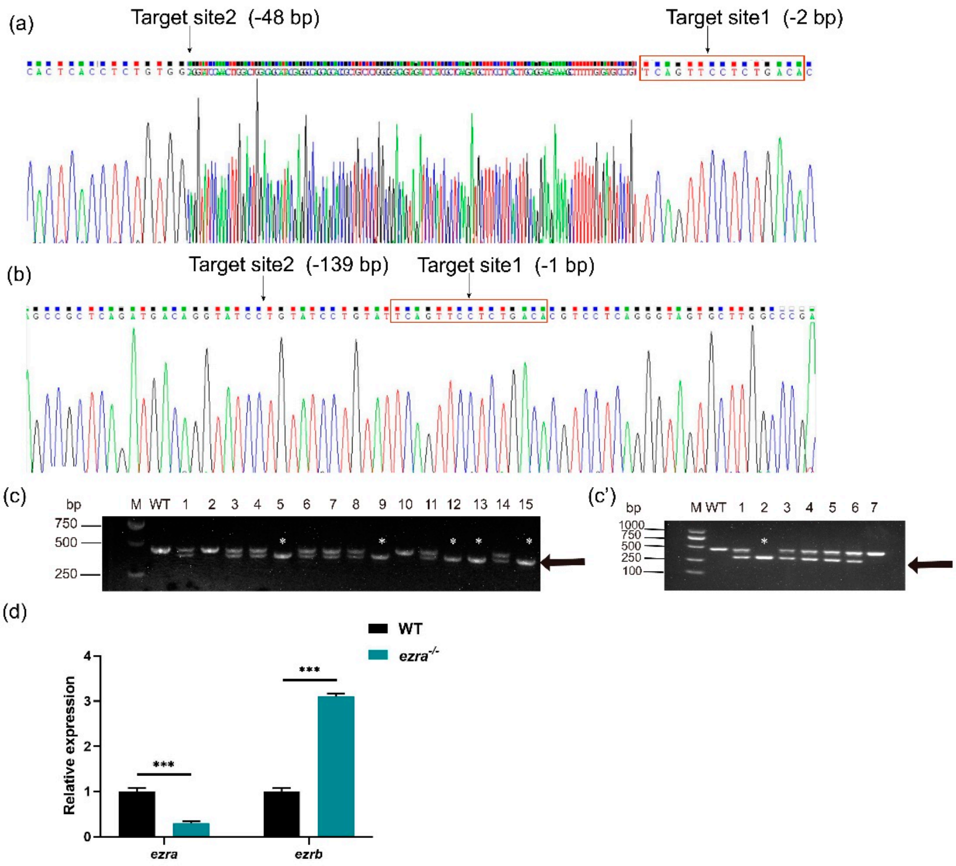

Figure S1 *ezra* Gene Knockout. (a,b) Sanger sequencing results of the *ezra*^{line1} mutant (a) and *ezra*^{line2} mutant (b). (c-c′) Genotyping results of *ezra*^{line1} F2 adult fish (c) and *ezra*^{line2} F2 adult fish (c′). M: DNA marker; black arrows indicate knockout bands. (d) mRNA expression levels of *ezra* and *ezrb* in *ezra*$^{-/-}$ embryos, *t*-test, ***: P<0.001. For RT-qPCR analyses, three independent biological replicates were prepared for both WT and *ezrin*-MO embryos, with each replicate consisting of 50 embryos at 48 hpf.

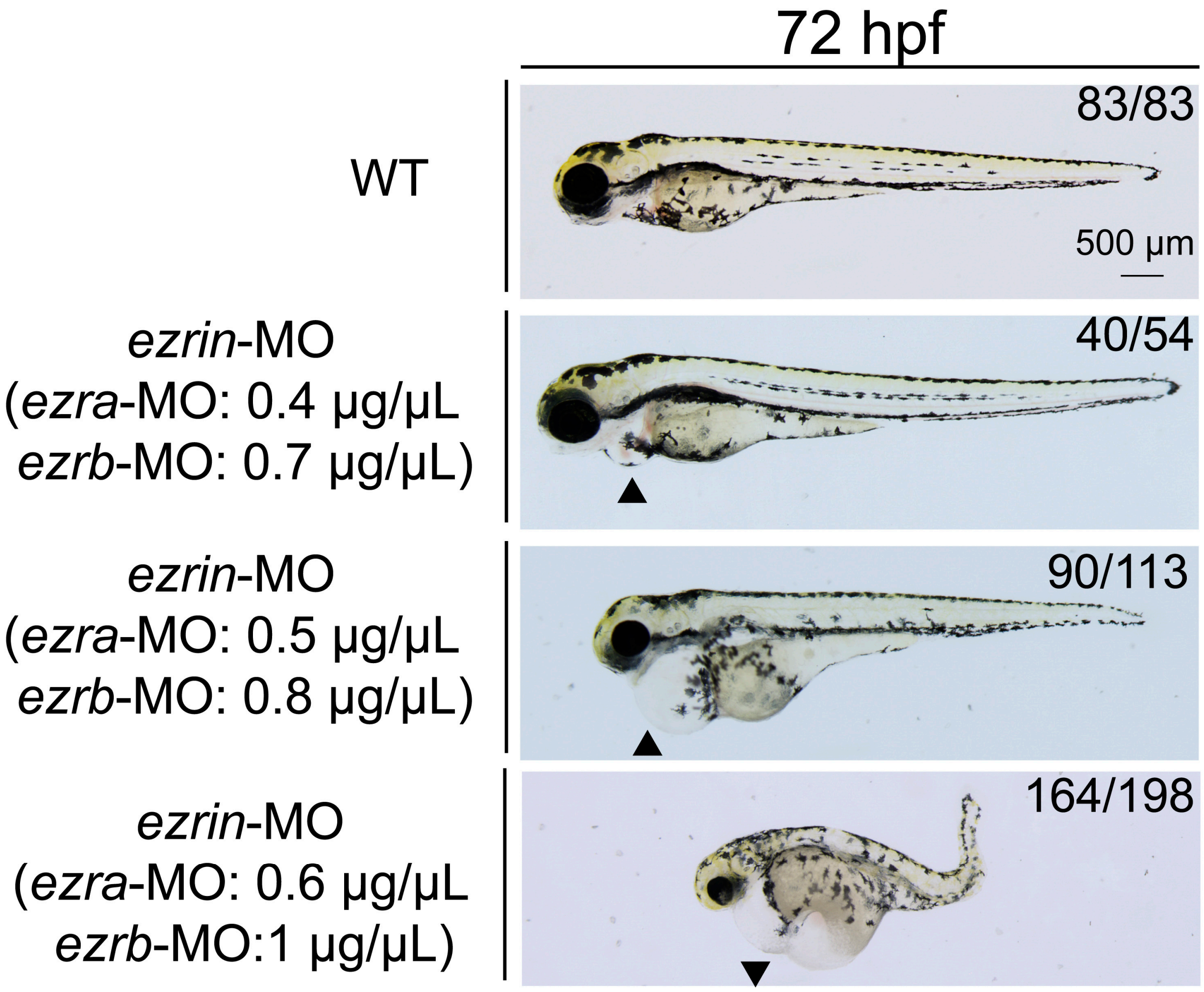

Figure S2 Morphological images of *ezrin*-MO embryos injected with different concentrations at 72 hpf.

Table S1 Primers of RT-qPCR experiment

Primers	Sequence (5' → 3')
ezra-qF	TTGCTGGAAGAAGCCAGGAG
ezra-qR	CTGCACCTCTTTAGCCCTGT
ezrb-qF	GTGGTGAAGACCATCGGGTT
ezrb-qR	ACGTCCTGAGAAGACACCTTT
vmhc-qF	GAGCTTGATGAGGCAGAAG
vmhc-qR	CAGCATAACGGAGACACAG
amhc-qF	CACCAGCAGACACTGGATG
amhc-qR	GCTCCAAGTCCATTCTGAC
tbx2b-qF	AACTGGCAGAGATGCTGGTC
tbx2b-qR	ACACCGGTCATTTTGGTGGT
bmp2b-qF	CTGCTGACCACAAGTTTTCG
*bmp*2b-qR	CAAAGACAGCAGCAATCCC
gata5-qF	CGCAAACCAAAGATGCCCAA
gata5-qR	AACTGTGTCGATGCCTGTGT
notch1b-qF	TCGTAAGCCCAGCACTAA

Primers	Sequence (5' → 3')
notch1b-qR	GTTTCCCTTTCCGTCTTG
bmp4-qF	GCCGTCGTACCACAGTATCT
bmp4-qR	CCTCCAGGTGCTCTTCATGA
has2-qF	GCCTCATCTGTGGAGATGGT
has2-qR	ATGCACTGAACACACCCAAA
VCANA-qF	TGTCACACAAACCCTTGTCGT
VCANA-qR	CCCCTGGAGACGACATTCAC
spp1-qF	AGCGACTACAAAAAATCCATCGTCT
spp1-qR	CTGAACAAGTTTGGCAGCAGTTCGA
atp1a3a-qF	CTGATCTTCGGGCTGTTTGAG
atp1a3a-qR	CCTTTTCAACCCAACCTCCTG
atp1b2b-qF	AACCCTGCATCATCATCAAGC
atp1b2b-qR	CCACATTGACGTCCTCATTCC
cacna1da-qF	ATGTGGTCCAGTGTGTGTTTG
cacna1da-qR	GATGTACGTTCCCTTGCACTC
cacna1fb-qF	CAGATTTCGAGCCTGGTGAAG
cacna1fb-qR	TCCATAATGACGGCGACAAAC
cacna2d2b-qF	CAGAATAAAGACCTGGGCACG
cacna2d2b-qR	CTCCCAAATGCAACCTCTACG
cacna2d3a-qF	ACCTCACCATTACAGCTCACA
cacna2d3a-qR	AAACTTCAGCCGCTCACATTT
cacna2d4a-qF	CAATCTGCCTGTCAATACGCA
cacna2d4a-qR	GTACCAGTTGCGATTCCTTCC
cacng2a-qR	TGTTCTTCTTGGAGTCGCTCT
cacng2a-qF	TGGTCTCTGTATAGCTGCCAG
cacng3b-qF	ACGAGGAAGTCATGACCCATT
cacng3b-qR	TAGGCCAGCAGAGACAAAGAA
cacng8a-qF	GGCCGTCAACATTTACATCGA
cacng8a-qR	ATTGGGAAGTGTGGCTACTGA
cox5b1-qF	CCAAACAAGACCCTCACATCG
cox5b1-qR	CTTCACCCTGATGCAACCAAA
slc8a4a-qF	GACGGGATGTTCACCAAGATG
slc8a4a-qR	CAGCTGCTCCATGTCTTTCTC
cdkn1a-q-R	TTCTGCTGCTTTTCCTGACAC
cdkn1a-q-F	ACTTTGCGTCTGAGAAACCAC
gadd45bb-qF	CGATGAGGAGGACGAGAATGA
gadd45bb-qR	TGCAGTAACTTCCCACTTCCT
slc8a4b-qF	TCTGCGGGCATAATTTTGAGG
slc8a4b-qR	GGAACGTCAATACAGCTTCCC
sesn3-qF	CACGACACGGAGAAGACAATC
sesn3-qR	AATACGTCAAATCACAGGCCG
mapk10-qF	GTCCTGGATGTGAAGATTGCC
mapk10-qR	AGCATGAGTCTGGTTCTGGAA
rac3a-qF	ATGTTATGGTGGATGGCAAGC
rac3a-qR	GTTAGGACAGTGATGGCGAAC
rac3b-qF	ATCAGCTACACCACAAATGCC
rac3b-qR	TCCCATAAGCCAAGGTTGACT
arrb1-qF	AAGAGCCTGACACGTCTACAA
arrb1-qR	TCTTCTGGCCCTGGTTGTAAT
mknk2b-qF	AATTTTGATTGTTCCGCCCGA

Primers	Sequence (5' → 3')
mknk2b-qR	TGTCCTGGCCTCTTCTCAATT
zak-qF	CACTTTCCTCCGCTGATCAAG
zak-qR	TTGCAGTCCTTTTGTCCTGTG
dusp2-qF	CCGGAAGAAAGACTCCACTCT
dusp2-qR	AGACATTGAGGACTGCTGTGA
dusp5-qF	GAACTTCCATGCCCATTACCC
dusp5-qR	ACAGGAAAGGCAAGATCTCCA
nfkbiaa-qF	ACCCTCAGCTTGTTGATCAGA
nfkbiaa-qR	GGGAAGGTAAGAATGGAACGC
parp4-qF	AAAGACACACACACTTCCTGC
parp4-qR	TTGAGGCTGATTCTGGACACT
parp3-qF	ATTCCTCATAACTTTGGCCGC
parp3-qR	TCAGCTTTAAGACTCTGGGCA
atp2b2-q-F	CACTCTCCTCCATCAGAGCAC
atp2b2-q-R	ACACCACATCCACTTCTCCAG
atp2b1b-q-F	CTGATGGAAGTTACCGCATGT
atp2b1b-q-R	CACCATTGCTTGTCAGGATTT
cacna1aa-q-F	CGGTCATGAGAGAGACAGAGG
cacna1aa-q-R	GGACCACAGGTGAGTAGGTGA
cacna1bb-q-F	GGCACTTTACAACGAATTGGA
cacna1bb-q-R	GATTTTGGCTGTGGTGTCTGT
cacna1e-q-F	TCACTAGAGGTGGGTTTGTGG
cacna1e-q-R	GCGCTTCATCAGTTTGTTCTC
plcd3b-q-F	TGCCATTTAGTGATGTGGTGA
plcd3b-q-R	GCAGTGGTTCTCCAGAGACAG
camk2d2-q-F	AAGGCTGGTGCCTATGATTTT
camk2d2-q-R	ATTGCACCCTTGAGTTTCCTT
camk1db-q-F	GAGATTGCAGTGCTGAGGAAG
camk1db-q-R	ATGACTGGGGCTCTCGTAAAT
nppa-q-F	CAGCAGACGGATGTACAAGC
nppa-q-R	CTGATGCCTCTTCTGTTGCC
hand2-q-F	AAACAGAGGCCTTCAAAGCG
hand2-q-R	TTCAGCTCCAATGCCCAAAC
fosab-qF	CTACAACCCGAGCTCTTACCC
fosab-qR	AAGCTGGTCAGTTTCAGCTTG
tnnt2a-qF	TCAGTGACCATCAGAAAACGTCA
tnnt2a-qR	TCAACAGTGGTCAGCTCCTC
tnni2b.1-qF	GACATCCACGTACAGCAGCC
tnni2b.1-qR	TGGAAGTCATCTTTTTCTCAGACAT
cnn1a-qF	CTTTGTGCGAGCCATTGGTG
cnn1a-qR	AAGCCTTTAGTCTTGGCCAGT
myhc4-qF	AGCTGCGTCGTGATCTTGAA
myhc4-qR	GATTGGCCTTTGCTTTGGCA
myh7l-qF	ATGGTAGAACGCAGGGATGC
myh7l-qR	TCTACGTGCCTCAGACTTCG
cmlc1-qF	AGAGCCCGAAGTGGACCT
cmlc1-qR	AAGGACGTGTAGTTAATGCAG

Table S2 WISH experiment probe primers

Primers	Sequence (5' → 3')
bmp4-ISH-F	CCACAGTATCTGCTCGACCTC
bmp4-ISH-R	GCGTAATACGACTCACTATAGGGATAGCGTGATTGGTGGAGTTG
notch1b-ISH-F	ATAGCGAGGAAGAGGAAGACG
notch1b-ISH-R	GCGTAATACGACTCACTATAGGGGAGTCGATCCATGTGGTCTGT
nppa-ISH-F	CAGCAGACGGATGTACAAGC
nppa-ISH-R	GCGTAATACGACTCACTATAGGGGGAAGACCCTATGCGATCCA
nkx2.5-ISH-F	CGGGACATACTGAACCTGGA
nkx2.5-ISH-R	GCGTAATACGACTCACTATAGGGTGCTGTTGGACTGTGAAGGT
hand2-ISH-F	GTCGCTGTCATGAAGAACCC
hand2-ISH-R	GCGTAATACGACTCACTATAGGGTTCAGCTCCAATGCCCAAAC
tnnt2a-ISH-F	CAGGAGGAAGAGCAAGTGGA
tnnt2a-ISH-R	GCGTAATACGACTCACTATAGGGTTTTGCATGTAACCGCCGAA
